# Identification of Tumor Suppressive *miR-144-5p* Targets: *FAM111B* Expression Accelerates the Malignant Phenotypes of Lung Adenocarcinoma

**DOI:** 10.3390/ijms25189974

**Published:** 2024-09-16

**Authors:** Yuya Tomioka, Naohiko Seki, Takayuki Suetsugu, Yoko Hagihara, Hiroki Sanada, Yusuke Goto, Naoko Kikkawa, Keiko Mizuno, Kentaro Tanaka, Hiromasa Inoue

**Affiliations:** 1 Department of Pulmonary Medicine, Graduate School of Medical and Dental Sciences, Kagoshima University, Kagoshima 890-8520, Japan; k4829264@kadai.jp (Y.T.); taka3741@m2.kufm.kagoshima-u.ac.jp (T.S.); k5382596@kadai.jp (Y.H.); k8173956@kadai.jp (H.S.); keim@m.kufm.kagoshima-u.ac.jp (K.M.); k9288090@kadai.jp (K.T.); inoue@m2.kufm.kagoshima-u.ac.jp (H.I.); 2 Department of Functional Genomics, Graduate School of Medicine, Chiba University, Chuo-ku, Chiba 260-8670, Japan; yusukegoto@chiba-u.jp (Y.G.); naoko-k@hospital.chiba-u.jp (N.K.)

**Keywords:** microRNA, *miR-144-5p*, tumor-suppressor, passenger strand, *FAM111B*, lung adenocarcinoma

## Abstract

Accumulating evidence suggests that the passenger strands microRNAs (miRNAs) derived from pre-miRNAs are closely involved in cancer pathogenesis. Analysis of our miRNA expression signature of lung adenocarcinoma (LUAD) and The Cancer Genome Atlas (TCGA) data revealed that *miR-144-5p* (the passenger strand derived from pre-*miR-144*) was significantly downregulated in LUAD tissues. The aim of this study was to identify therapeutic target molecules controlled by *miR-144-5p* in LUAD cells. Ectopic expression assays demonstrated that *miR-144-5p* attenuated LUAD cell aggressiveness, e.g., inhibited cell proliferation, migration and invasion abilities, and induced cell cycle arrest and apoptotic cells. A total of 18 genes were identified as putative cancer-promoting genes controlled by *miR-144-5p* in LUAD cells based on our in silico analysis. We focused on a family with sequence similarity 111 member B (*FAM111B*) and investigated its cancer-promoting functions in LUAD cells. Luciferase reporter assay showed that expression of *FAM111B* was directly regulated by *miR-144-5p* in LUAD cells. *FAM111B* knockdown assays showed that LUAD cells significantly suppressed malignant phenotypes, e.g., inhibited cell proliferation, migration and invasion abilities, and induced cell cycle arrest and apoptotic cells. Furthermore, we investigated the *FAM111B*-mediated molecular networks in LUAD cells. Identifying target genes regulated by passenger strands of miRNAs may aid in the discovery of diagnostic markers and therapeutic targets for LUAD.

## 1. Introduction

Cancer is not only one of the most serious life-threatening diseases, but it can also pose a huge burden to society in any country. Lung cancer has the highest incidence and mortality rate of any cancer, with an estimated 2.3 million cases diagnosed and 1.8 million deaths in 2021 [1]. Histologically, lung cancer is divided into two groups: small cell lung cancer (SCLC), which accounts for 15% of lung cancer patients, and non-small cell lung cancer (NSCLC), which accounts for 85% of lung cancer patients. Lung adenocarcinoma (LUAD) accounts for approximately 60% of patients with NSCLC [2].

Although surgical resection is a curative treatment for lung cancer patients, many patients have advanced stages of the disease at the time of diagnosis, and their life prognosis is extremely poor, with only 20% of patients surviving 5 years [3]. The prognosis for LUAD patients with advanced stage has been improved significantly with the development of molecular-targeted drugs and immune checkpoint inhibitors [4,5].

One of the serious clinical problems among LUAD is brain metastasis. The incidence of this complication is particularly pronounced in LUAD with activating driver gene mutations, e.g., *EGFR*, *ALK*, and *ROS-1* [6]. Discovery of new therapeutic target molecules is an important research topic to improve the prognosis of LUAD patients.

MicroRNAs (miRNAs) are single-stranded short non-coding RNAs (19–22 nucleotides in length), and they act as fine controller of gene expression and modulate almost all biological processes, e.g., proliferation, cell cycle control, programmed cell death, differentiation, invasiveness [7,8]. Regulation of miRNAs is essential for maintaining normal cellular function. Accumulating evidence demonstrated that aberrant expressed miRNAs caused to disrupt RNA networks, and these events have been implicated in the development, metastasis, and drug resistance of cancer cells [9,10].

A unique feature of miRNA is that a single miRNA controls a vast number of RNA transcripts in normal and disease cells. Therefore, it is possible to trace the RNA networks controlled by miRNAs within cells starting with a miRNA interest.

Based on the miRNA expression signature of LUAD, we focused on aberrant expressed miRNAs, and to investigate the functional significance and their controlled genes closely involved in the molecular pathogenesis of LUAD [11].

Our RNA-sequence-based signature revealed that some passenger strand miRNAs were significantly downregulated in LUAD tissues [11]. In miRNA biogenesis, two single-stranded miRNAs (the guide strand and the passenger strand) are derived from the miRNA precursor. Previous concepts suggested that only the guide strand of a miRNA actually functions to regulate the miRNA’s target RNAs within the cell. On the other hand, the passenger strand was thought to be degraded within the cell and to be non-functional [12]. Therefore, passenger strands derived from miRNA precursors have been left behind in cancer research and their functions remain poorly understood.

To date, *miR-144-3p* (the guide strand derived from pre-*miR-144*) has been shown to be an antitumor miRNA in various types of cancers, including lung cancer. In the analysis of our signature, we focused on *miR-144-5p* (the passenger strand), because its expression was significantly downregulated in LUAD tissues, and the role of *miR-144-5p* has not been fully elucidated in LUAD cells. Antitumor roles of *miR-144-5p* in LUAD cells was confirmed by our functional assays.

Interestingly, a total of 18 cell cycle-related genes (*ARHGAP11A*, *CDC3*, *CENPF*, *CENPN*, *CHEK1*, *CP*, *DEPDC1B*, *ECT2*, *FAM111B*, *FAM64A*, *HELLS*, *HJURP*, *KIF11*, *NCAPG*, *RALGPS2*, *SGOL1*, *SPC24*, and *TRIP13*) were identified as putative *miR-144-5p* controlled genes, and potential therapeutic targets for this disease. The analysis of the poorly characterized passenger strand of miRNAs may reveal new therapeutic targets for LUAD. Our RNA-sequence-based miRNA expression signatures provide the research field with information about which miRNAs they need to analyze.

## 2. Results

### 2.1. Genomics Structure of miR-144-5p and miR-144-3p, and Their Expression in LUAD Clinical Specimens

**Figure 1:**
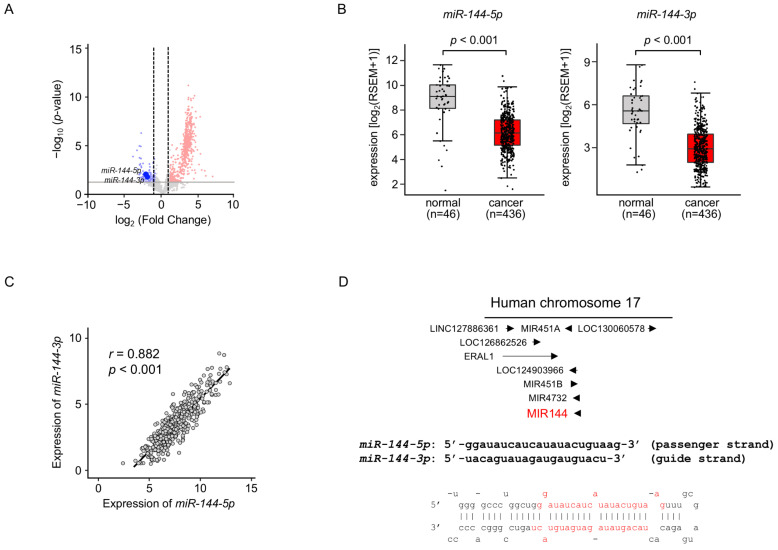
Expression levels of *miR-144-5p* and *miR-144-3p* in LUAD clinical specimens (**A**) Volcano plot showing the miRNA expression signature obtained through miRNA sequencing (GEO accession number: GSE230229). The log_2_ fold change (FC) in expression is plotted on the x-axis and the log_10_ *p*-value is on the y-axis. The red and blue dots represent the upregulated (log_2_ FC > 1.0 and *p* < 0.05) miRNAs and downregulated (log_2_ FC < −1.0 and *p* < 0.05), respectively. (**B**) Validation of *miR-144-5p* and *miR-144-3p* expression levels in LUAD clinical specimens. The expression levels of both miRNAs were markedly reduced in cancer tissues. (*p* < 0.001). (**C**) Positive correlations (Spearman’s rank test) between the expression levels of *miR-144-5p* and *miR-144-3p* in clinical specimens (*r* = 0.882, *p* < 0.001). (**D**) The chromosomal position of pre-*miR-144* within the human genome. The mature sequences of *miR-144-5p* (passenger strand) and *miR-144-3p* (guide strand) are shown.

We previously created the miRNA expression signature of LUAD based on RNA-sequencing [11]. Our signature revealed that both strands of pre-*miR-144* (*miR-144-5p*: the passenger strand and *miR-144-3p*: the guide strand) were downregulated in LUAD tissues (Figure 1A). Downregulation of both strands of miRNAs in LUAD clinical specimens was confirmed by a large number of cohort data by TCGA datasets (*p* < 0.001; Figure 1B). Spearman’s rank analysis revealed a positive correlation between the expression levels of *miR-144-5p* and *miR-144-3p* (*r* = 0.882, *p* < 0.001; Figure 1C).

Human genome database showed that pre-*miR-144* was located on chromosome 17q11.2. Interestingly, four miRNAs (*miR-451a*, *miR-451b*, *miR-144*, and *miR-4732*) are located in close proximity in this region (Figure 1D). Among these miRNAs, the downregulation of *miR-451a* was detected in TCGA database analysis.

Our recent studies demonstrated that some passenger strands of miRNAs are closely involved in the molecular pathogenesis of human cancers [13,14]. In this study, we focused on *miR-144-5p* and investigated its functional role to identify target genes in LUAD cells.

This section may be divided by subheadings. It should provide a concise and precise description of the experimental results, their interpretation, as well as the experimental conclusions that can be drawn.

### 2.2. Antitumor Roles of miR-144-5p in LUAD Cells

**Figure 2:**
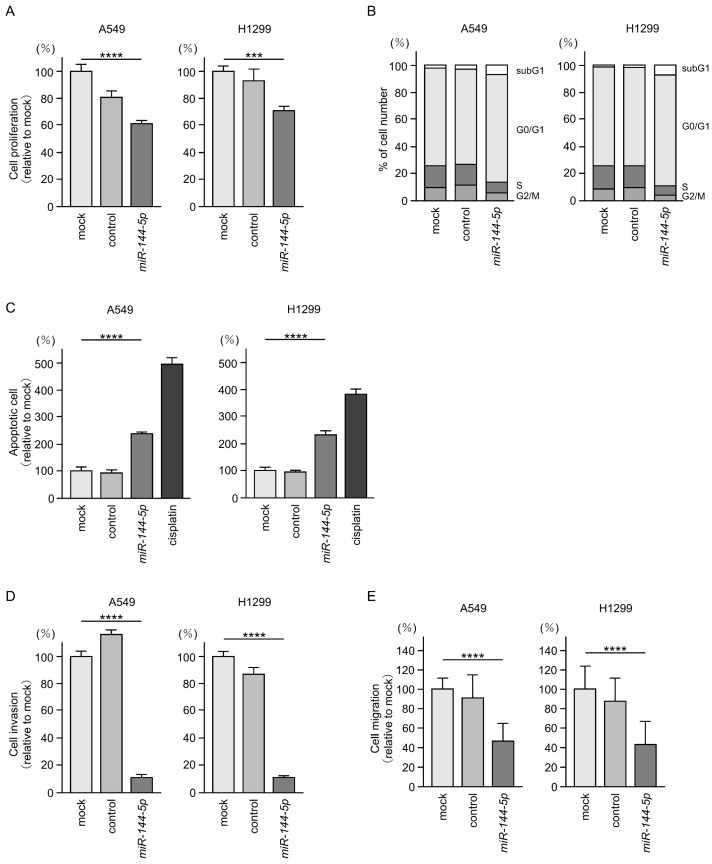
Antitumor functions of *miR-144-5p* in LUAD cells (A549 and H1299). (**A**) Cell proliferation was evaluated using XTT assay. Cancer cell viability was analyzed 72 h after transient transfection of miRNAs. (**B**) At 72 h after transient transfection with *miR-144-5p*, cell cycle status evaluated using flow cytometry. (**C**) At 72 h after transient transfection with *miR-144-5p*, apoptotic cells was evaluated using flow cytometry with Annexin V-FITC- and PI-PerCP-Cy5-5-A-stained cells. Cisplatin (30 µM) was used as a positive control for induction of apoptosis. (**D**) At 72 h after seeding *miR-144-5p*-transfected cells into the chambers, cell invasion was evaluated using Matrigel invasion assays. (**E**) At 72 h after seeding *miR-144-5p* transfected cells into the chambers, cell migration assessed using a membrane culture system. ***, *p* < 0.001; ****, *p* < 0.0001.

Antitumor effects of *miR-144-5p* were assessed by ectopic expression assays using two LUAD cell lines (A549 and H1299). Expression of *miR-144-5p* inhibited the proliferation of LUAD cells (Figure 2A). Flow cytometry analysis revealed that *miR-144-5p* expression induced G0/G1 arrest in LUAD cells (Figure 2B). Furthermore, we observed an increase in apoptotic cells by *miR-144-5p* expression (Figure 2C).

Cancer cell invasion and migration abilities were markedly suppressed after *miR-144-5p* expression in LUAD cells (Figure 2D,E). Typical images of the invasion and migration assays after *miR-144-5p* expression are shown in Figure S1.

Our present data strongly suggest that *miR-144-5p* acts as an antitumor miRNA in LUAD cells.

### 2.3. Identification of miR-144-5p Controlled Cancer-Promoting Genes in LUAD Cells

The next area of interest is determining which genes are controlled by antitumor *miR-144-5p* in LUAD cells.

**Figure 3:**
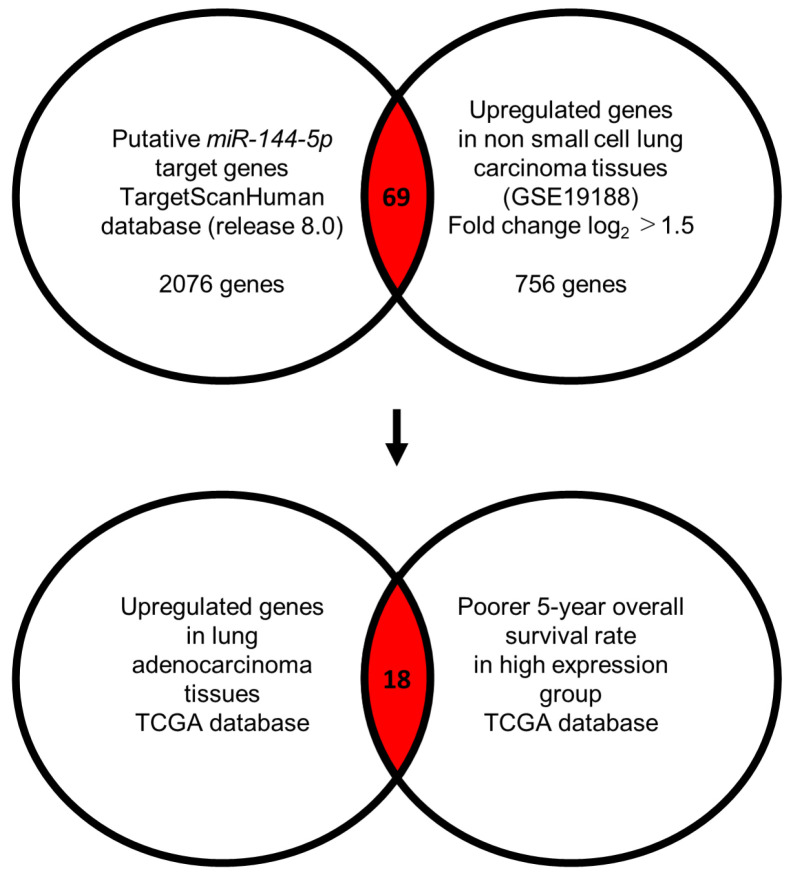
Flowchart for identification of *miR-144-5p* targets in LUAD cell. To identify putative targets of *miR-144-5p* in LUAD cells, we used two datasets: the TargetScanHuman database (release 8.0) and our original mRNA expression profile (Upregulated genes in non-small cell lung carcinoma tissues; GEO accession number: GSE19188). A total of 69 genes were identified as candidate targets of *miR-144-5p*. Furthermore, we searched for genes that were associated with the prognosis of LUAD patients using two databases: OncoLnc (http://www.oncolnc.org, accessed on 17 May 2024) and GEPIA (http://gepia2.cancer-pku.cn/#analysis, accessed on 17 May 2024). Among the *miR-144-5p* target genes, 18 genes were upregulated in LUAD tissues, and closely associated with poor prognosis in LUAD patients.

Our strategy for the identification of *miR-144-5p* controlled genes in LUAD cells is shown in Figure 3. The TargetScanHuman database (release 8.0) revealed that 2076 genes contained *miR-144-5p* binding sites within their 3′ untranslated regions (UTR). Using the gene expression profile with the GEO database (accession number: GSE19188), we identified 756 genes that were upregulated (log_2_ fold change > 1.5) in NSCLC tissues compared to normal tissues. Integrating two datasets revealed that a total of 69 genes were identified as putative *miR-144-5p* controlled genes in LUAD cells (Table 1).

### 2.4. Clinical Significance of miR-144-5p Controlled Genes by TCGA-LUAD Analysis

We used the TCGA-LUAD database to confirm the clinical significance of 69 genes potentially controlled by *miR-144-5p*.

**Figure 4:**
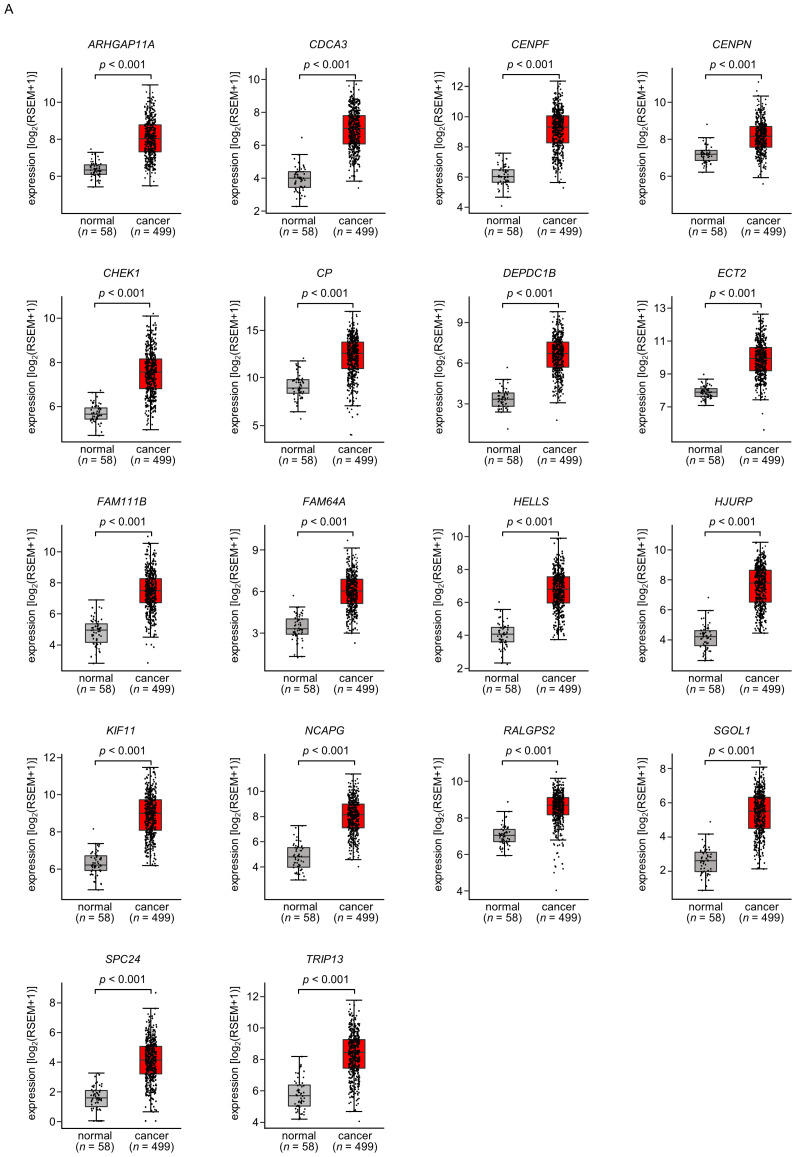

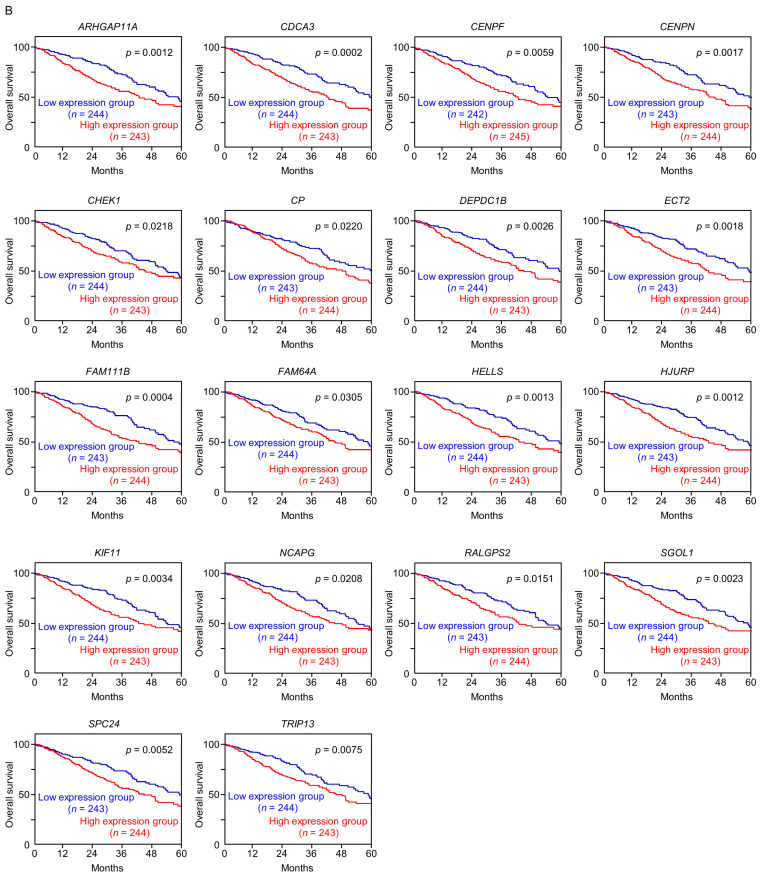
Expression levels and 5-year overall survival rate of the 18 target genes regulated by *miR-144-5p* in LUAD (**A**) The expression levels of the 18 target genes of *miR-144-5p* (*ARHGAP11A*, *CDCA3*, *CENPF*, *CENPN*, *CHEK1*, *CP*, *DEPDC1B*, *ECT2*, *FAM111B*, *FAM64A*, *HELLS*, *HJURP*, *KIF11*, *NCAPG*, *RALGPS*, *SGOL1*, *SPC24*, *TRIP13*) in LUAD clinical specimens were assessed using the TCGA-LUAD dataset. All genes were upregulated in LUAD tissues (*n* = 499) compared with normal tissues (*n* = 58) (*p* < 0.001). (**B**) Kaplan–Meier curves of the 5-year overall survival rates based on expression of the 18 target genes (*ARHGAP11A*, *CDCA3*, *CENPF*, *CENPN*, *CHEK1*, *CP*, *DEPDC1B*, *ECT2*, *FAM111B*, *FAM64A*, *HELLS*, *HJURP*, *KIF11*, *NCAPG*, *RALGPS*, *SGOL1*, *SPC24*, *TRIP13*) are shown. Lower expression levels of all 18 genes were significantly associated with poorer overall survival in LUAD patients. The patients (*n* = 487) were divided into high and low-expression groups based on the median gene expression level. The red and blue lines denote the high and low expression groups, respectively.

A total of 18 of these target genes (Table 1, Bold) were upregulated in LUAD tissues (*n* = 499) compared with normal lung tissues (*n* = 58) (Figure 4A), and closely associated with poor prognosis in LUAD patients (5-year overall survival rate, *p* < 0.05) (Figure 4B).

Among these genes, the expression of two genes (*CDCA3*: *p* = 0.0002 and *FAM111B*: *p* = 0.0004) had a significant impact on the prognosis of lung cancer patients. Recently, it has been reported that *CDCA3* is regulated by *miR-144-5p* in lung cancer cells [15]. Therefore, in this analysis, we focused on *FAM111B* and conducted further analysis.

### 2.5. Direct Regulation of FAM111B by miR-144-5p in LUAD Cells

**Figure 5:**
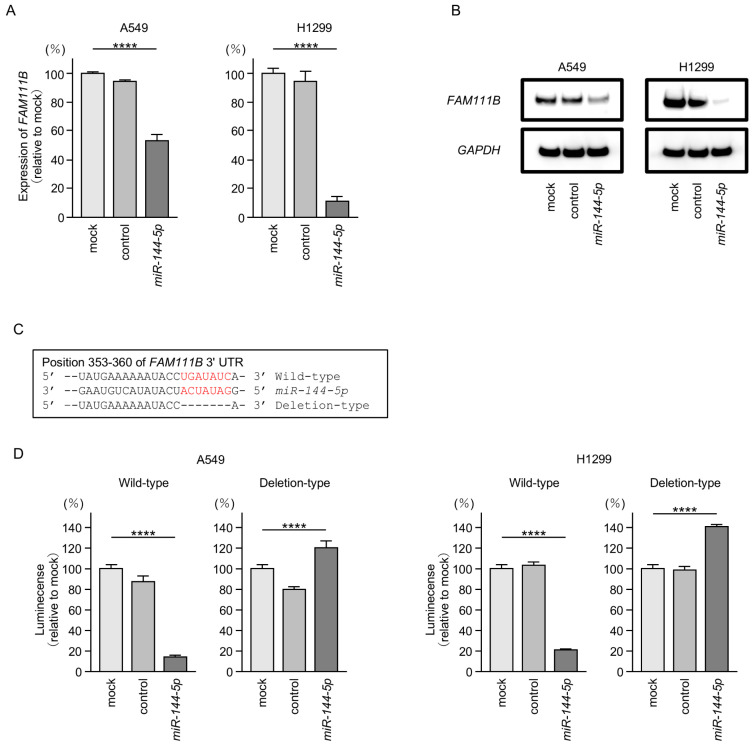
*MiR-144-5p* expression directly regulated *FAM111B* in LUAD cells. (**A**) Expression level of *FAM111B* mRNA is markedly reduced by ectopic expression of *miR-144-5p* in LUAD cells (A549 and H1299). Total RNA was isolated 72 h after miRNA transfection and quantified by real-time PCR. *GAPDH* was used as an internal control. (**B**) Significant reduction of the FAM111B protein level by ectopic expression of *miR-144-5p* in LUAD cells (A549 and H1299). Proteins were isolated 72 h after *miR-144-5p* transfection and quantified by Western blotting. GAPDH was used as an internal control. (**C**) Putative *miR-144-5p* binding sites in the 3′UTR of the *FAM111B* gene were detected using the TargetScanHuman database (release 8.0). (**D**) Dual luciferase reporter assays revealed reduced luminescence activity after co-transfection of *miR-144-5p* with a vector containing the *miR-144-5p* binding site (wild-type) in LUAD cells (A549 and H1299). In contrast, no luminescence activity was observed after co-transfection of *miR-144-5p* with a vector lacking the *miR-144-5p* binding site (deletion-type) in LUAD cells. ****, *p* < 0.0001.

Both mRNA and protein expression levels were significantly reduced by ectopic expression of *miR-144-5p* in LUAD cells (Figure 5A,B). Full-size images of Western blots are shown in Figure S2.

Subsequently, we demonstrated, using the luciferase reporter assay, that *miR-144-5p* directly binds to the 3′UTR of the *FAM111B* gene. The putative *miR-144-5p*-binding site on the 3′-UTR of the *FAM111B* gene is shown in Figure 5C. Luciferase activity was markedly decreased when LUAD cells were co-transfected with *miR-144-5p* and a vector containing a *miR-144-5p*-binding sequence (Figure 5D). In contrast, no decrease in luciferase activity was observed when a vector lacking the *miR-144-5p*-binding sequence was used (Figure 5D). These results indicated *that miR-144-5p* directly binds to the 3′-UTR of *FAM111B* and modulates its expression in LUAD cells.

### 2.6. Functional Significance of FAM111B in LUAD Cells

**Figure 6:**
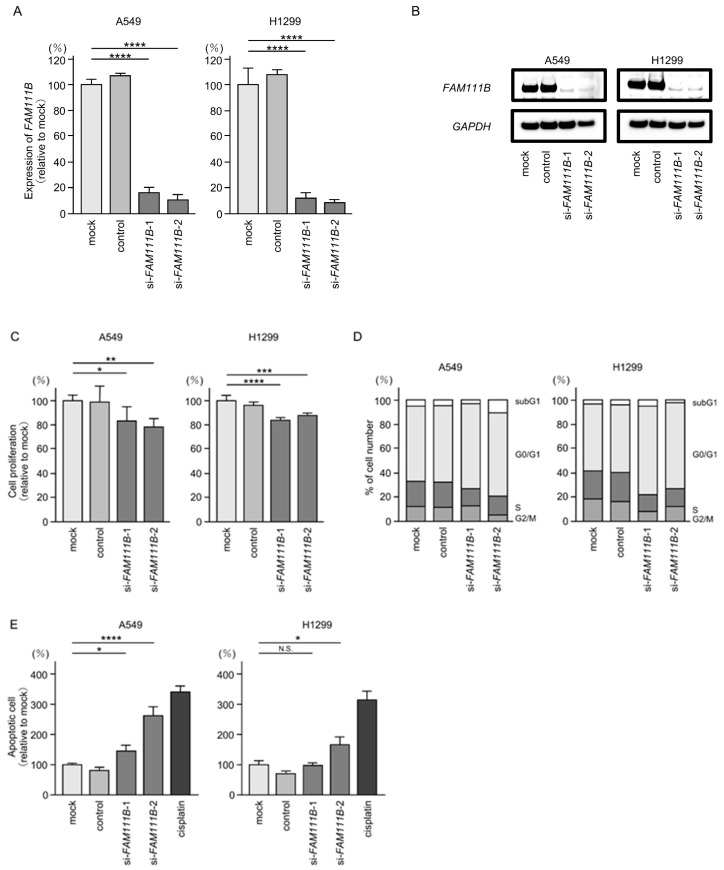

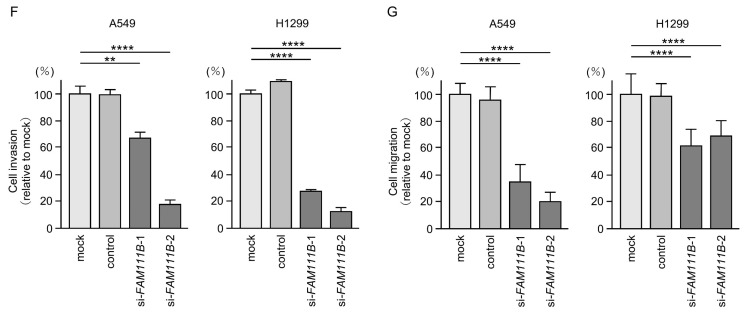
Effects of knockdown of *FAM111B* by siRNAs in LUAD cells (A549 and H1299) (**A**) The inhibitory effects of two different siRNAs targeting *FAM111B* (si*FAM111B*-1 and si*FAM111B*-2) expression were examined. *FAM111B*- mRNA levels were effectively inhibited by each siRNA in LUAD cells (A549 and H1299). (**B**) FAM111B protein levels were effectively inhibited by two siRNAs (si*FAM111B*-1 and si*FAM111B*-2) in LUAD cells (A549 and H1299). (**C**) Cell proliferation was evaluated using XTT assays 72 h after siRNA transfection into LUAD cells. (**D**) At 72 h after transient transfection with si*FAM111B*-1 and si*FAM111B*-2, cell cycle status was evaluated using flow cytometry. (**E**) At 72 h after transient knockdown of *FAM111B*, apoptotic cells were evaluated using flow cytometry with Annexin V-FITC- and PI-PerCP-Cy5-5-A-stained cells. Cisplatin (30 µM) was used as a positive control for induction of apoptosis. (**F**) At 72 h after seeding *FAM111B*-knockdown cells into the chambers, cell invasion assessed using Matrigel invasion assays. (**G**) At 72 h after seeding *FAM111B*-knockdown cells into the chambers, cell migration was assessed using a membrane culture system. *, *p* < 0.05; **, *p* < 0.01; ***, *p* < 0.001; ****, *p* < 0.0001; N.S., not significant.

We investigated the oncogenic function of *FAM111B* in LUAD cells using siRNA-mediated *FAM111B* knockdown assays. Both mRNA and protein levels were significantly reduced by two siRNAs (si*FAM111B*-1 and si*FAM111B*-2) in LUAD cells (Figure 6A,B). Full-size images of the Western blots are shown in Figure S3.

Cancer cell proliferation was slightly inhibited by siRNAs-transfected LUAD cells. In H1299 cells, there was only a slight effect on cell proliferation (Figure 6C). Moreover, cell cycle arrest (G0/G1 phase), and induced apoptotic cells were detected in siRNAs-transfected LUAD cells (Figure 6D,E). However, there was only a slight increase in apoptotic cells in H1299 cells. Cancer cell invasion and migration abilities were markedly suppressed in siRNAs-transfected LUAD cells (Figure 6F,G). Typical images of invasion and migration assays in siRNAs-transfection LUAD cells are shown in Figure S4.

### 2.7. Clinical Significance of FAM111B in LUAD Clinical Specimens

**Figure 7:**
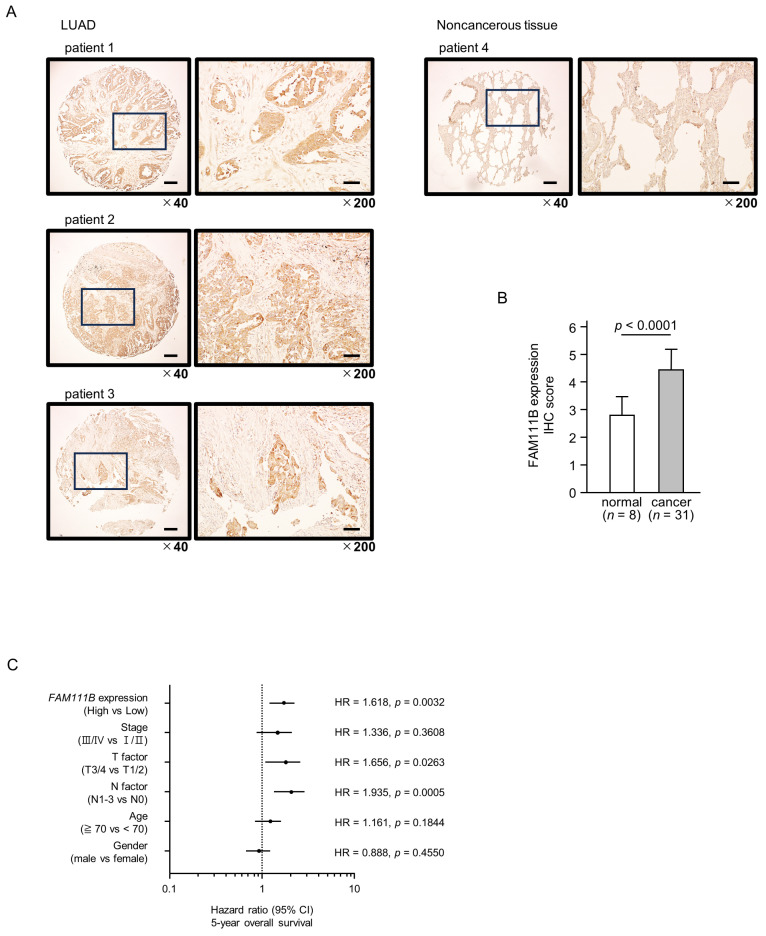

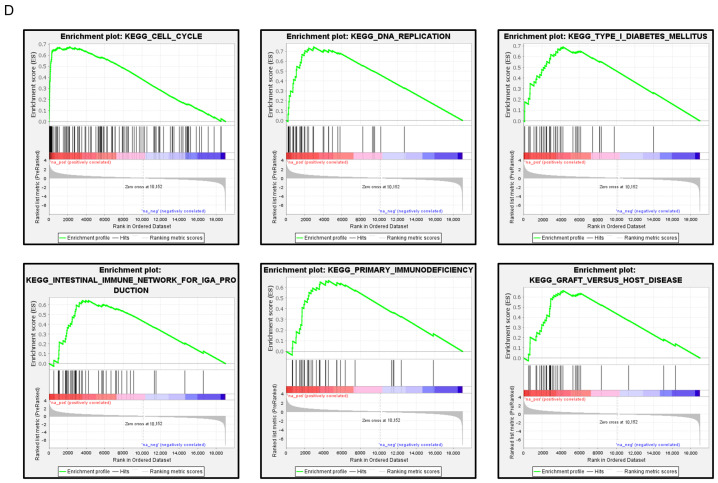
*FAM111B* expression and its clinical significance in LUAD. (**A**) Immunohistochemical staining of FAM111B. (**B**) Cancer tissues showed strong immunostaining, in contrast to the weak staining observed in noncancerous tissues. The data are means and standard errors of the means. Mann–Whitney U-tests. Scale bar: 200 µm (low magnification); 50 µm (high magnification). (**C**) Forest plot showing the results of multivariate Cox proportional hazards regression analysis of the 5-year overall survival rate. A significantly lower overall survival rate was observed in patients with high *FAM111B* expression. The data were sourced from TCGA-LUAD datasets. (**D**) FAM111B-mediated pathways identified by gene set enrichment analysis. The “cell cycle”, “DNA replication” pathways were enriched in patients with high *FAM111B* expression.

To confirm the expression of *FAM111B* in LUAD clinical specimens, immunostaining was conducted. Stronger immunostaining of the FAM111B protein was observed in cancerous tissues than in normal lung tissues. (Figure 7A). The protein expression of FAM111B was scored, and expression of FAM111B in cancerous tissues was significantly higher than in normal tissues (Figure 7B). The characteristics of patient samples used for immunostaining are shown in Table S1.

Multivariate analysis revealed that *FAM111B* expression is an independent prognostic factor for LUAD, even when accounting for clinical prognostic factors including stage, T-factor, N-factor, age, and gender (Figure 7C). Specifically, higher *FAM111B* expression correlated with a reduced 5-year overall survival rate.

To identify *FAM111B*-mediated molecular pathways in LUAD patients, we performed gene set enrichment analysis with TCGA-LUAD data. The “cell cycle”, “DNA replication” pathways were enriched in patients with high *FAM111B* expression compared to low *FAM111B* expression (Table 2, Figure 7D).

## 3. Discussion

RNA-sequence based miRNA expression signatures suggest that two types of miRNAs (the guide strands and the passenger strands) derived from miRNA precursors are deeply involved in the molecular pathogenesis of human cancer [16]. Based on previous concepts of miRNA biogenesis, analysis of the guide strands of miRNAs has been prioritized in cancer research. On the other hand, recent research have revealed that some passenger strands of miRNAs also control the expression of target molecules within cells, and dysregulate passenger strands act as oncogenes and tumor-suppressors in cancer cells [16,17]. Exploring the RNA networks controlled by passenger strands might uncover new therapeutic targets for cancer.

In the human genome, *miR-144* is located close to *miR-451a*, *miR-451b*, and *miR-4732* on the chromosome 17q11.2 [18]. Previous studies demonstrated that expression of *miR-451a* was frequently downregulated in multiple type of cancers, including lung cancers [19,20,21]. Gain-of-function assays showed ectopic expression of *miR-451a* attenuated malignant phenotypes of lung cancer cells via targeting several oncogenes [22,23]. Recent studies indicated that both strands of *miR-4732-5p* and *miR-4732-3p* prevented lung cancer aggressiveness through inhibited TBX15/TNFSF11 axis or PI3K/Akt/GSK3β/Snail pathway [24,25].

Numerous studies have demonstrated that *miR-144-3p* (the guide strand) has tumor suppressor functions in various types of cancers, including lung cancer [26]. Compared to the analysis of *miR-144-3p*, *miR-144-5p* (the passenger strand) has not been fully characterized in cancer cells. Previously, the expression of *miR-144-5p* enhanced radio sensitivity of NSCLC cells in vitro and mouse xenografts in vivo through targeting activating transcription factor 2 [27]. Recent studies have demonstrated that aberrantly expressed non-coding RNAs or circulating RNAs may be involved in promoting cancer cell malignant transformation by adsorbing tumor-suppressive miRNAs [28,29]. Circular RNA, *circRACGAP1* was overexpressed in NSCLC tissues, and its downregulation enhanced the Gefitinib sensitivity of NSCLC cells [30]. *CircRACGAP1* was directly regulated by *miR-144-5p* in NSCLC cells [30].

Apart from our current data, it has been shown that *miR-144-5p* acted as an antitumor miRNA in lung cancer cells. Taking into account previous reports, it has been shown that suppression of expression of miRNA cluster (*miR-451*/*miR-144*/*miR-4732*) located on chromosomes 17q11.2 had a profound impact on the malignant transformation of lung cancer cells. An important task for the future will be to clarify the molecular mechanisms underlying the expression control of the miRNAs present in this cluster.

Our next challenge is to identify therapeutic targets for LUAD among the molecules regulated by these clustered miRNAs. In this study, a total of 18 genes were identified as LUAD therapeutic targets controlled by *miR-144-5p*. Among these targets, we have already analyzed three genes (*FAM64A*; *miR-99a* target, *HELLs*; *miR-150-3p* target, and *TRIP13*; *miR-139-3p* target) as oncogenic target molecules regulated by tumor-suppressive miRNAs in lung cancer [13,14,31]. Treatment of DCZ0415 (*TRIP13* specific inhibitor) significantly attenuated the malignant transformation of LUAD cells. Moreover, when combined with anticancer drugs (cisplatin and carboplatin), DCZ0415 exerted a synergistic effect in inhibiting cell proliferation [14].

In this study, we focused on *FAM111B* because its oncogenic roles in LUAD cells are not fully understood. Previous studies have demonstrated that the *FAM111B* protein contains a trypsin/cysteine protease-like domain at its C-terminus [32]. However, the function of this domain in cells is not fully understood. According to several reports, *FAM111B* is a poorly characterized protease involved in DNA repair, cell cycle regulation, and apoptosis [32].

Regarding human disease, *FAM111B* gene mutations have been reported in patients with POIKTMP, a hereditary multisystemic fibrosis disorder. The syndrome is characterized by fibrosis of multiple organs, including the skin and lungs [32]. However, the molecular mechanism by which *FAM111B* mutations cause POIKTMP remains unknown.

In human cancers, overexpression of *FAM111B* has been reported in multiple cancer types, e.g., esophageal cancer, hepatocellular carcinoma, bladder cancer, ovarian cancer, breast cancer and lung cancer [33,34,35,36,37,38]. Furthermore, functional analysis of *FAM111B* suggested that aberrant expression of *FAM111B* had cancer-promoting functions such as promoting the cell cycle progression and inhibition of apoptosis [33,34,35,36,37,38]. Our siRNA-mediated knockdown assays demonstrated that downregulation of *FAM111B* significantly inhibited cancer cell proliferation, migration, invasion abilities, and induced cell cycle arrest and apoptosis, and strongly suggested as a cancer-promoting gene in LUAD cells. A Recent study of hepatoma cells showed that silencing *FAM111B* induced cell cycle arrest (G0/G1), and reduced cell migration and invasion abilities [39]. Importantly, *FAM111B* reduced the p53 expression level by degrading p53 protein in hepatoma cells [39]. Inactivation of the p53 by *FAM111B* led to activation of the cyclin D1-CDK4/6 pathway, thereby inducing cell proliferation [39]. In LUAD cells (*KRAS*-driven LUAD under serum-starvation conditions), *FAM111B*-knockout cells demonstrated that *FAM111B* controlled cell cycle progression in a cyclin D1-CDK4-dependent manner by degrading p16 [40]. These reports suggest that *FAM111B* is involved in cancer cell proliferation by controlling gatekeeper genes in cell cycle regulation.

## 4. Materials and Methods

### 4.1. Analysis of LUAD Clinical Specimens by TCGA Database

Expression of miRNA and miRNA target genes in LUAD tissues assessed using the following databases: The Cancer Genome Atlas (TCGA) (https://www.cancer.gov/tcga, accessed on 17 May 2024), Genomic Data Commons Data Portal (https://portal.gdc.cancer.gov/, accessed on 17 May 2024), and FIREBROWSE (http://firebrowse.org/, accessed on 17 May 2024). Overall survival data were obtained from OncoLnc (http://www.oncolnc.org/) (data downloaded on 17 May 2024) and cBioPortal (https://www.cbioportal.org/, accessed on 17 May 2024).

### 4.2. Functional Assays of miRNAs and miRNA Target Genes in LUAD Cells

Two LUAD cell lines, A549 and H1299 were used in this study; two cell lines were obtained from American Type Culture Collection (Manassas, VA, USA).

The procedures for RNA extraction and qRT-PCR were described in our previous studies [11,14,22].

Functional assays (e.g., proliferation, cell cycle, apoptosis, migration and invasion) were performed for transient transfection of small RNAs (miRNAs and siRNAs) into LUAD cells. All miRNA precursors were transfected at 10 nM, and all siRNAs were transfected at 5nM into cell lines using Opti-MEM (catalog no.: 31985070, Gibco, Carlsbad, CA, USA) and Lipofectamine RNAiMAX (Invitrogen, Carlsbad, CA, USA). The analysis procedures have been described in our previous studies [11,14,22].

The reagents used in the experiments are shown in the Table S2.

### 4.3. Identification of Oncogenic Targets Controlled by miR-144-5p in LUAD Cells

To identify oncogenic targets controlled by *miR-144-5p* in LUAD, we used TargetScanHuman v8.0 (https://www.targetscan.org/vert_80/, accessed on 24 May 2023), and a gene expression profile from the GEO database (GEO accession number: GSE19188). We used GeneCodis 4 software to infer the molecular functions of the *miR-144-5p* target genes [41]. Gene set enrichment analysis software was used to infer the molecular pathways controlled by these genes [42,43].

### 4.4. Plasmid Construction and Dual-Luciferase Reporter Assay

Vector construction and dual-luciferase reporter assays were performed as described in our previous studies [11,14,22]. The purified plasmid vectors were transfected into LUAD cells using Lipofectamine 2000 (Invitrogen) at 20 ng/well. The vector insertion sequences are shown in Figure S5, and the reagents used are listed in Table S2.

### 4.5. Western Blotting and Immunohistochemistry

Western blotting and immunohistochemical analysis were performed according to our previous studies [11,14,22]. The intensity and area of staining were evaluated based on previous reports [44], and immunohistochemical scores were calculated. The antibodies used in the study are listed in Table S2. A list of clinical specimens evaluated by immunohistochemistry is given in Table S1.

### 4.6. Statistical Analysis

Statistical analyzes were achieved using R ver. 4.4.0 (R Core Team, Vienna, Austria; https://www.R-project.org/, accessed on 25 April 2024) and GraphPad Prism 8 (GraphPad Software, La Jolla, CA, USA). The differences between the two groups were analyzed by Student’s *t*-tests. Multiple group comparison was performed using a one-way analysis of variance (ANOVA) and Tukey’s tests for post hoc analysis. Survival rates were analyzed by Kaplan–Meier survival curves and the log-rank test.

## 5. Conclusions

Our miRNA signature and TCGA-LUAD database analysis revealed that *miR-144-5p* (the passenger strand) was significantly downregulated in LUAD tissues. Ectopic expression of *miR-144-5p* attenuated the malignant phenotypes of LUAD cells, suggesting that this miRNA acted as an antitumor miRNA in LUAD cells. In total, 18 genes (*ARHGAP11A*, *CDC3*, *CENPF*, *CENPN*, *CHEK1*, *CP*, *DEPDC1B*, *ECT2*, *FAM111B*, *FAM64A*, *HELLS*, *HJURP*, *KIF11*, *NCAPG*, *RALGPS2*, *SGOL1*, *SPC24*, and *TRIP13*) were identified as therapeutic targets by *miR-144-5p* regulation in LUAD cells. *FAM111B* was directly regulated by *miR-144-5p*, and its overexpression facilitated LUAD cell aggressiveness. The involvement of the passenger strand in the molecular pathogenesis of LUAD, and the search for its regulatory genes are effective strategies for discovering therapeutic target for LUAD.

## Figures and Tables

**Table 1:** Putative target genes regulated by *miR-144-5p* in A549 cells.

Gene ID	Gene Symbol	Gene Name	*miR-144-5p* Total Sites	GSE19188 Log_2_ FC
139322	*APOOL*	apolipoprotein O-like	1	1.60
9824	***ARHGAP11A***	Rho GTPase activating protein 11A	2	1.64
9048	*ARTN*	artemin	1	2.24
29028	*ATAD2*	ATPase family, AAA domain containing 2	1	2.25
55971	*BAIAP2L1*	BAI1-associated protein 2-like 1	1	1.59
83461	***CDCA3***	cell division cycle associated 3	1	2.97
1063	***CENPF***	centromere protein F	1	2.98
55839	***CENPN***	centromere protein N	1	2.18
1111	***CHEK1***	checkpoint kinase 1	1	2.76
1281	*COL3A1*	collagen, type III, alpha 1	1	1.96
1356	***CP***	ceruloplasmin (ferroxidase)	1	1.60
200407	*CREG2*	cellular repressor of E1A-stimulated genes 2	1	1.51
55789	***DEPDC1B***	DEP domain containing 1B	1	3.26
79962	*DNAJC22*	DnaJ (Hsp40) homolog, subfamily C, member 22	1	2.03
1825	*DSC3*	desmocollin 3	1	2.49
667	*DST*	dystonin	1	2.84
1894	***ECT2***	epithelial cell transforming sequence 2 oncogene	1	2.23
85465	*EPT1*	ethanolaminephosphotransferase 1 (CDP-ethanolamine-specific)	1	1.82
374393	***FAM111B***	family with sequence similarity 111, member B	1	2.08
139231	*FAM199X*	family with sequence similarity 199, X-linked	1	1.53
10447	*FAM3C*	family with sequence similarity 3, member C	1	1.54
54478	***FAM64A***	family with sequence similarity 64, member A	1	2.71
83416	*FCRL5*	Fc receptor-like 5	1	2.20
2244	*FGB*	fibrinogen beta chain	1	1.89
10690	*FUT9*	fucosyltransferase 9 (alpha (1,3) fucosyltransferase)	2	1.53
2575	*GAGE1*	G antigen 1	1	1.66
163351	*GBP6*	guanylate binding protein family, member 6	2	1.76
9615	*GDA*	guanine deaminase	1	1.94
2877	*GPX2*	glutathione peroxidase 2 (gastrointestinal)	1	3.58
26585	*GREM1*	gremlin 1, DAN family BMP antagonist	1	4.10
8908	*GYG2*	glycogenin 2	3	1.55
3070	***HELLS***	helicase, lymphoid-specific	2	3.15
8357	*HIST1H3H*	histone cluster 1, H3h	1	1.70
55355	***HJURP***	Holliday junction recognition protein	1	3.63
8091	*HMGA2*	high mobility group AT-hook 2	2	2.83
3174	*HNF4G*	hepatocyte nuclear factor 4, gamma	2	1.71
3239	*HOXD13*	homeobox D13	1	1.55
3664	*IRF6*	interferon regulatory factor 6	2	1.50
3696	*ITGB8*	integrin, beta 8	1	1.64
3832	***KIF11***	kinesin family member 11	1	2.48
3798	*KIF5A*	kinesin family member 5A	1	1.68
79944	*L2HGDH*	L-2-hydroxyglutarate dehydrogenase	2	1.73
389421	*LIN28B*	lin-28 homolog B (C. elegans)	1	1.66
51678	*MPP6*	membrane protein, palmitoylated 6 (MAGUK p55 subfamily member 6)	1	1.66
64151	***NCAPG***	non-SMC condensin I complex, subunit G	1	2.83
132299	*OCIAD2*	OCIA domain containing 2	1	1.51
4986	*OPRK1*	opioid receptor, kappa 1	1	1.51
5080	*PAX6*	paired box 6	1	1.60
5122	*PCSK1*	proprotein convertase subtilisin/kexin type 1	1	2.53
51050	*PI15*	peptidase inhibitor 15	1	1.67
5865	*RAB3B*	RAB3B, member RAS oncogene family	1	3.16
55103	***RALGPS2***	Ral GEF with PH domain and SH3 binding motif 2	1	2.00
26575	*RGS17*	regulator of G-protein signaling 17	1	2.28
116832	*RPL39L*	ribosomal protein L39-like	1	1.99
5268	*SERPINB5*	serpin peptidase inhibitor, clade B (ovalbumin), member 5	1	4.25
151648	***SGOL1***	shugoshin-like 1 (S. pombe)	1	2.16
51804	*SIX4*	SIX homeobox 4	1	2.00
204962	*SLC44A5*	solute carrier family 44, member 5	1	3.07
169166	*SNX31*	sorting nexin 31	1	1.79
147841	***SPC24***	SPC24, NDC80 kinetochore complex component	1	2.18
23213	*SULF1*	sulfatase 1	1	2.44
6857	*SYT1*	synaptotagmin I	1	2.06
9066	*SYT7*	synaptotagmin VII	1	1.73
7036	*TFR2*	transferrin receptor 2	1	1.86
130827	*TMEM182*	transmembrane protein 182	1	1.57
9319	***TRIP13***	thyroid hormone receptor interactor 13	2	2.53
221806	*VWDE*	von Willebrand factor D and EGF domains	1	2.21
8840	*WISP1*	WNT1 inducible signaling pathway protein 1	1	1.72
8838	*WISP3*	WNT1 inducible signaling pathway protein 3	1	1.88

The bold indicates the 18 genes from Section 2.4.

**Table 2:** *FAM111B*-mediated pathways by Gene Set Enrichment Analysis (GSEA).

Pathway	Enrichment Score	Normalized Enrichment Score	*p*-Value	FDR
KEGG_CELL_CYCLE	0.67	2.49	<0.001	<0.001
KEGG_DNA_REPLICATION	0.74	2.25	<0.001	<0.001
KEGG_TYPE_I_DIABETES_MELLITUS	0.69	2.07	<0.001	<0.001
KEGG_INTESTINAL_IMMUNE_NETWORK_FOR_ IGA_PRODUCTION	0.64	2.01	<0.001	0.001
KEGG_PRIMARY_IMMUNODEFICIENCY	0.66	2.00	<0.001	0.001
KEGG_GRAFT_VERSUS_HOST_DISEASE	0.66	1.97	<0.001	0.001

## Data Availability

Publicly available datasets were analyzed in this study. These data can be accessed here: https://www.ncbi.nlm.nih.gov/geo/query/acc.cgi?acc=GSE230229 (accessed on 24 May 2024) and https://www.ncbi.nlm.nih.gov/geo/query/acc.cgi?acc=GSE19188 (accessed on 24 May 2024).

## Supplementary Materials

The following supporting information can be downloaded at: https://www.mdpi.com/article/10.3390/ijms25189974/s1.
