# Supplementary material for: Identification of Tumor Suppressive miR-144-5p Targets: FAM111B Expression Accelerates the Malignant Phenotypes of Lung Adenocarcinoma
Source: Int J Mol Sci. 2024 Sep 16;25(18):9974. doi: 10.3390/ijms25189974 (PMC11432174; doi:10.3390/ijms25189974)
Supplement: Supplementary file 1 [file ijms-25-09974-s001.zip › figure Supplement.pptx]

## Slide 1
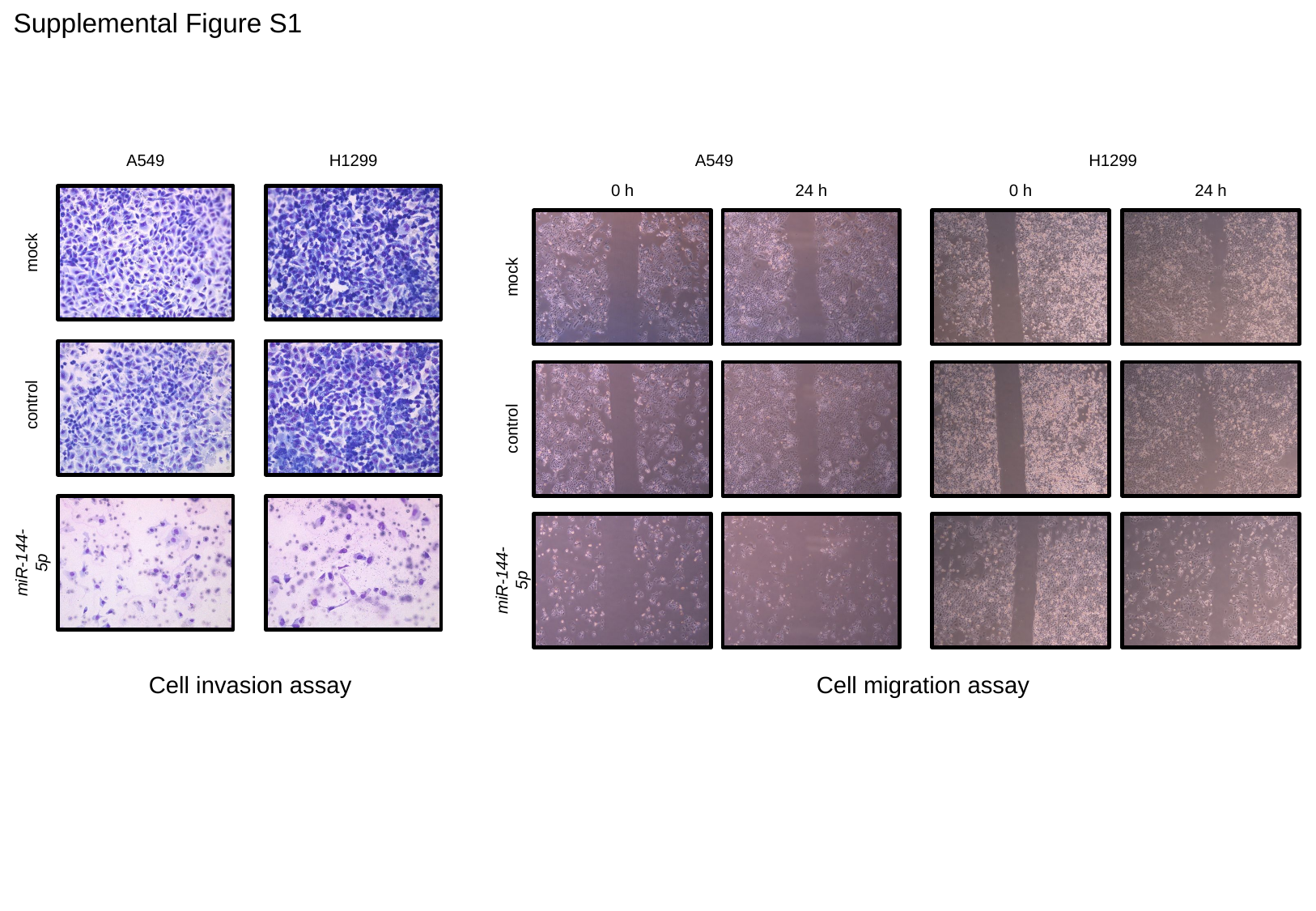

Supplemental Figure S1
A549
H1299
0 h
24 h
0 h
24 h
mock
control
miR-144-5p
A549
H1299
mock
control
miR-144-5p
Cell invasion assay
Cell migration assay

## Slide 2
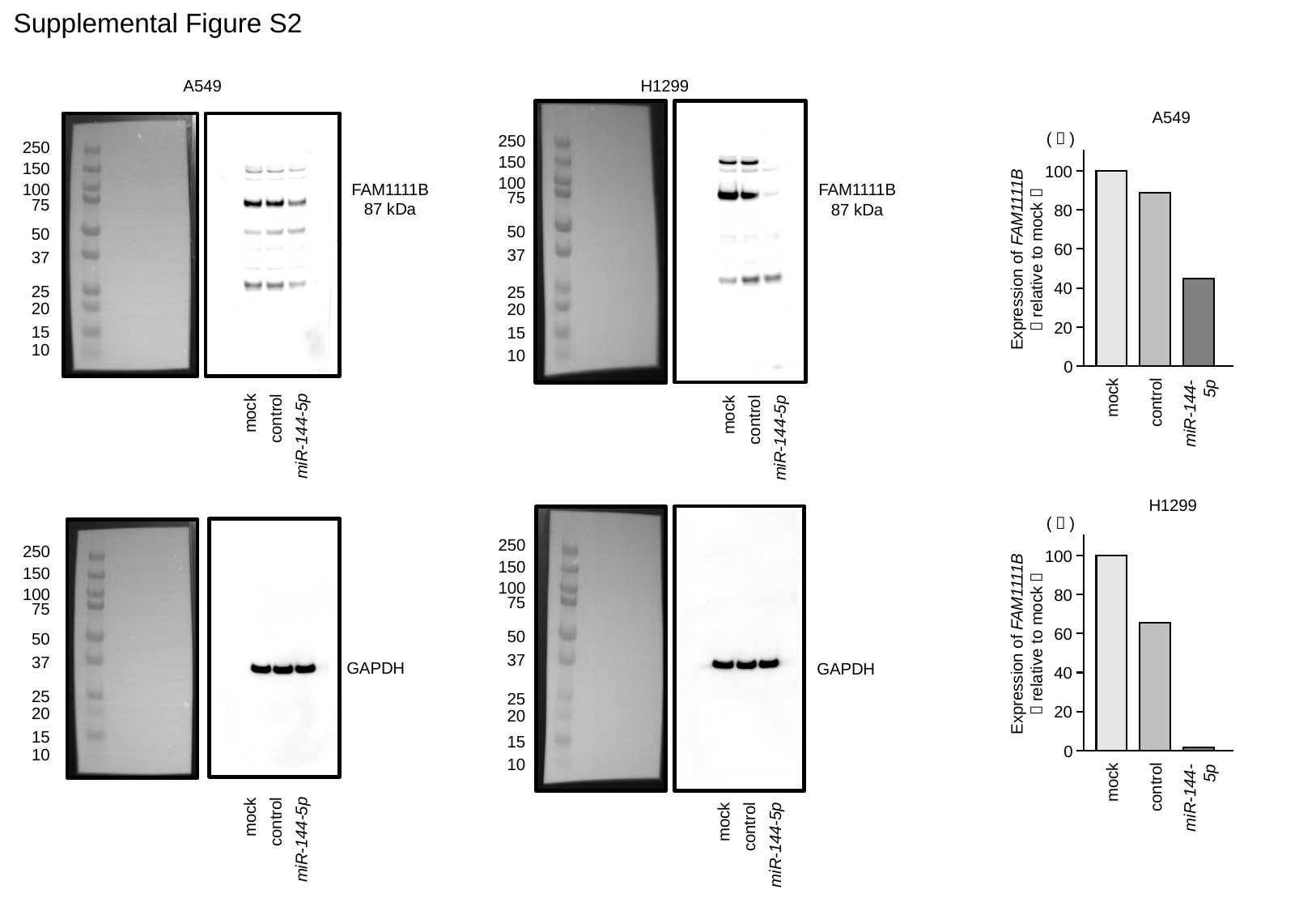

Supplemental Figure S2
A549
H1299
A549
(％)
250
250
150
150
100
100
100
FAM1111B
87 kDa
FAM1111B
87 kDa
75
75
80
50
50
60
Expression of FAM1111B
（relative to mock）
37
37
40
25
25
20
20
20
15
15
10
10
0
mock
control
miR-144-5p
mock
control
miR-144-5p
mock
control
miR-144-5p
H1299
(％)
250
250
100
150
150
100
100
80
75
75
60
Expression of FAM1111B
（relative to mock）
50
50
37
37
GAPDH
GAPDH
40
25
25
20
20
20
15
15
0
10
10
mock
control
miR-144-5p
mock
control
miR-144-5p
mock
control
miR-144-5p

## Slide 3
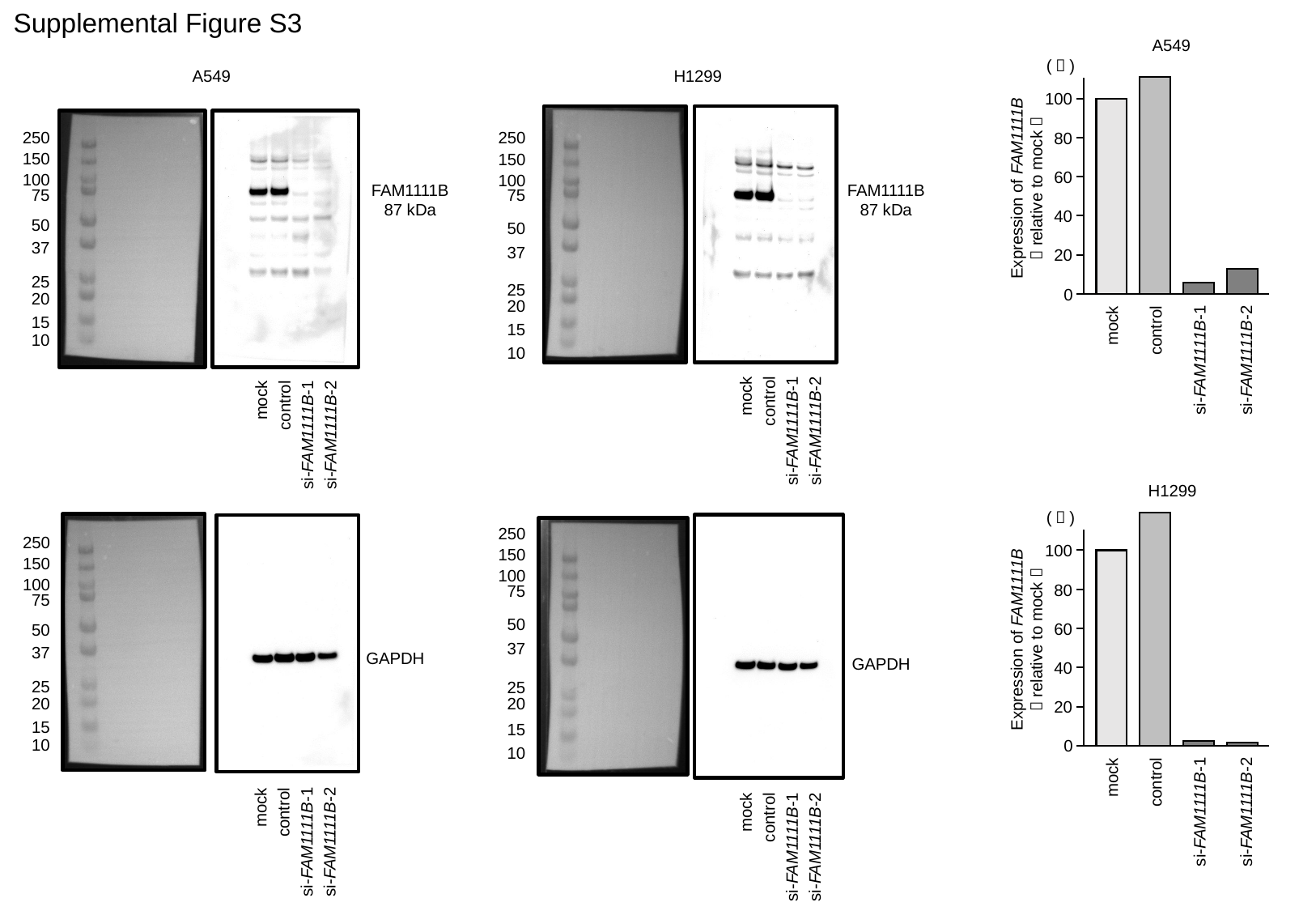

Supplemental Figure S3
A549
(％)
A549
H1299
100
250
250
80
150
150
60
Expression of FAM1111B
（relative to mock）
100
100
FAM1111B
87 kDa
FAM1111B
87 kDa
75
75
40
50
50
37
37
20
25
25
0
20
20
mock
control
si-FAM1111B-1
si-FAM1111B-2
15
15
10
10
mock
control
si-FAM1111B-1
si-FAM1111B-2
mock
control
si-FAM1111B-1
si-FAM1111B-2
H1299
(％)
250
250
100
150
150
100
100
80
75
75
50
60
50
Expression of FAM1111B
（relative to mock）
37
37
GAPDH
GAPDH
40
25
25
20
20
20
15
15
10
0
10
mock
control
si-FAM1111B-1
si-FAM1111B-2
mock
control
si-FAM1111B-1
si-FAM1111B-2
mock
control
si-FAM1111B-1
si-FAM1111B-2

## Slide 4
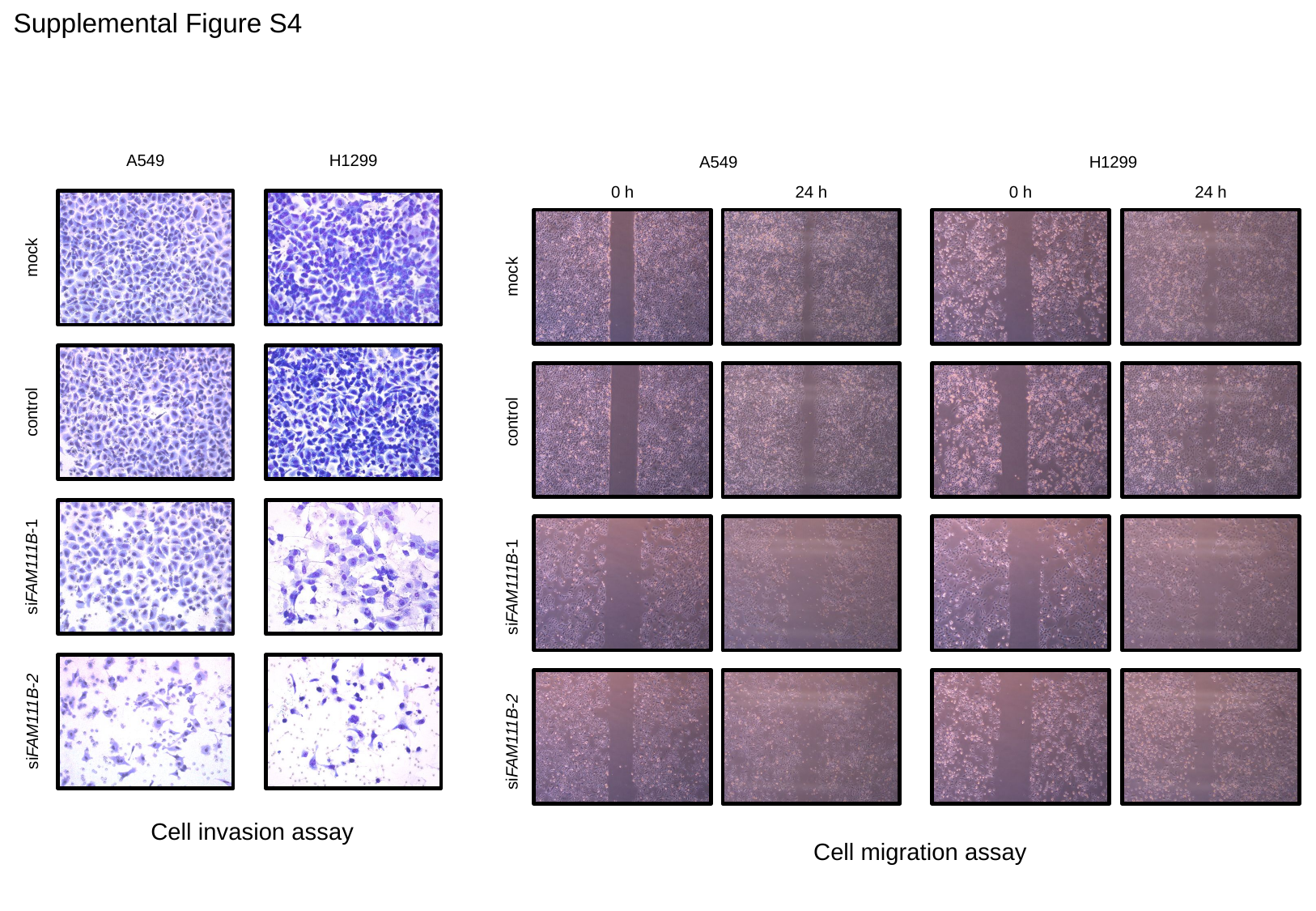

Supplemental Figure S4
A549
H1299
A549
H1299
0 h
24 h
0 h
24 h
mock
mock
control
control
siFAM111B-1
siFAM111B-1
siFAM111B-2
siFAM111B-2
Cell invasion assay
Cell migration assay

## Slide 5
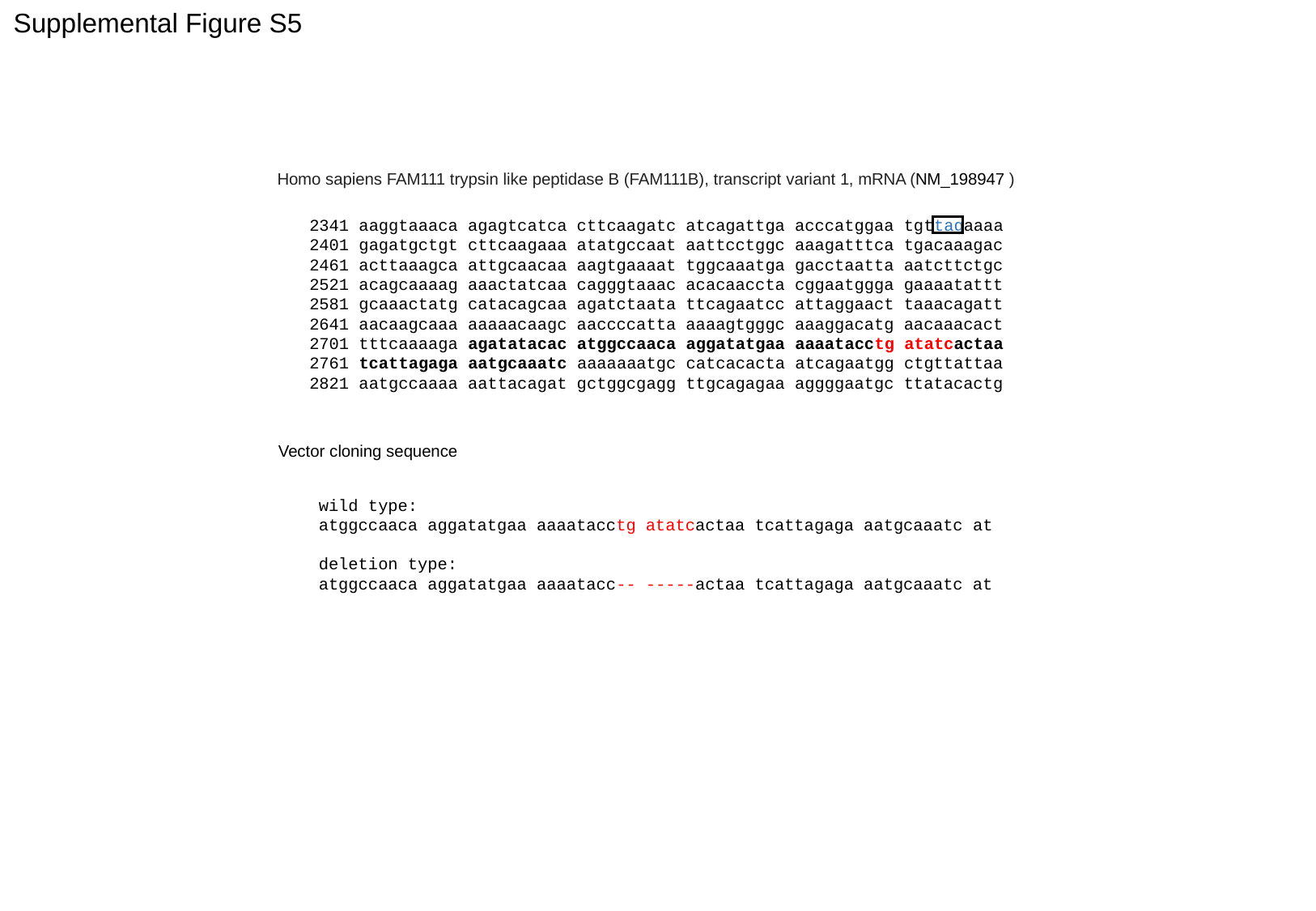

Supplemental Figure S5
Homo sapiens FAM111 trypsin like peptidase B (FAM111B), transcript variant 1, mRNA (NM_198947 )
 2341 aaggtaaaca agagtcatca cttcaagatc atcagattga acccatggaa tgttagaaaa
 2401 gagatgctgt cttcaagaaa atatgccaat aattcctggc aaagatttca tgacaaagac
 2461 acttaaagca attgcaacaa aagtgaaaat tggcaaatga gacctaatta aatcttctgc
 2521 acagcaaaag aaactatcaa cagggtaaac acacaaccta cggaatggga gaaaatattt
 2581 gcaaactatg catacagcaa agatctaata ttcagaatcc attaggaact taaacagatt
 2641 aacaagcaaa aaaaacaagc aaccccatta aaaagtgggc aaaggacatg aacaaacact
 2701 tttcaaaaga agatatacac atggccaaca aggatatgaa aaaatacctg atatcactaa
 2761 tcattagaga aatgcaaatc aaaaaaatgc catcacacta atcagaatgg ctgttattaa
 2821 aatgccaaaa aattacagat gctggcgagg ttgcagagaa aggggaatgc ttatacactg
Vector cloning sequence
wild type:
atggccaaca aggatatgaa aaaatacctg atatcactaa tcattagaga aatgcaaatc at
deletion type:
atggccaaca aggatatgaa aaaatacc-- -----actaa tcattagaga aatgcaaatc at

## Slide 6
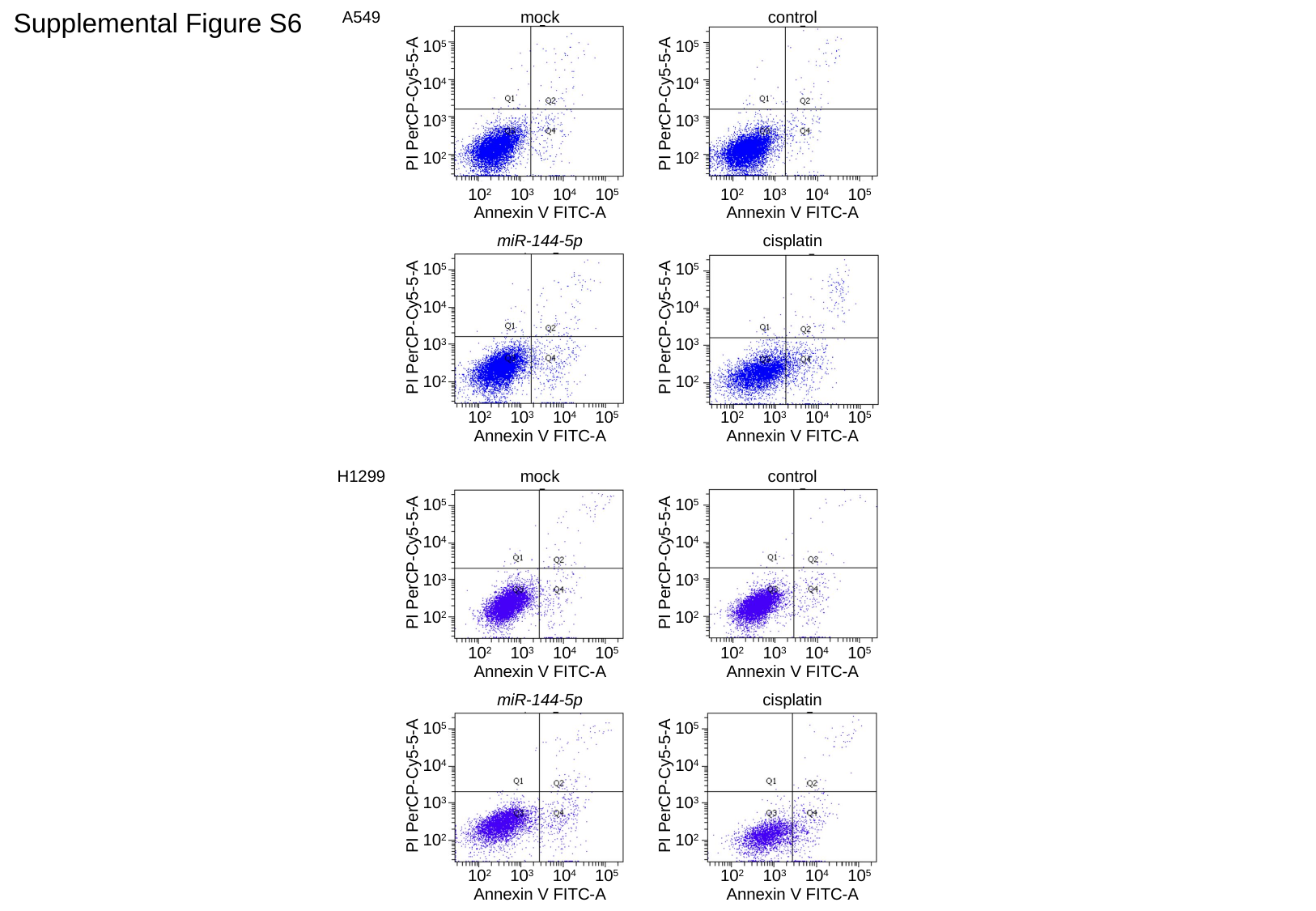

Supplemental Figure S6
A549
mock
105
104
PI PerCP-Cy5-5-A
103
102
102
103
104
105
Annexin V FITC-A
control
105
104
PI PerCP-Cy5-5-A
103
102
102
103
104
105
Annexin V FITC-A
miR-144-5p
105
104
PI PerCP-Cy5-5-A
103
102
102
103
104
105
Annexin V FITC-A
cisplatin
105
104
PI PerCP-Cy5-5-A
103
102
102
103
104
105
Annexin V FITC-A
H1299
mock
105
104
PI PerCP-Cy5-5-A
103
102
102
103
104
105
Annexin V FITC-A
control
105
104
PI PerCP-Cy5-5-A
103
102
102
103
104
105
Annexin V FITC-A
miR-144-5p
105
104
PI PerCP-Cy5-5-A
103
102
102
103
104
105
Annexin V FITC-A
cisplatin
105
104
PI PerCP-Cy5-5-A
103
102
102
103
104
105
Annexin V FITC-A

## Slide 7
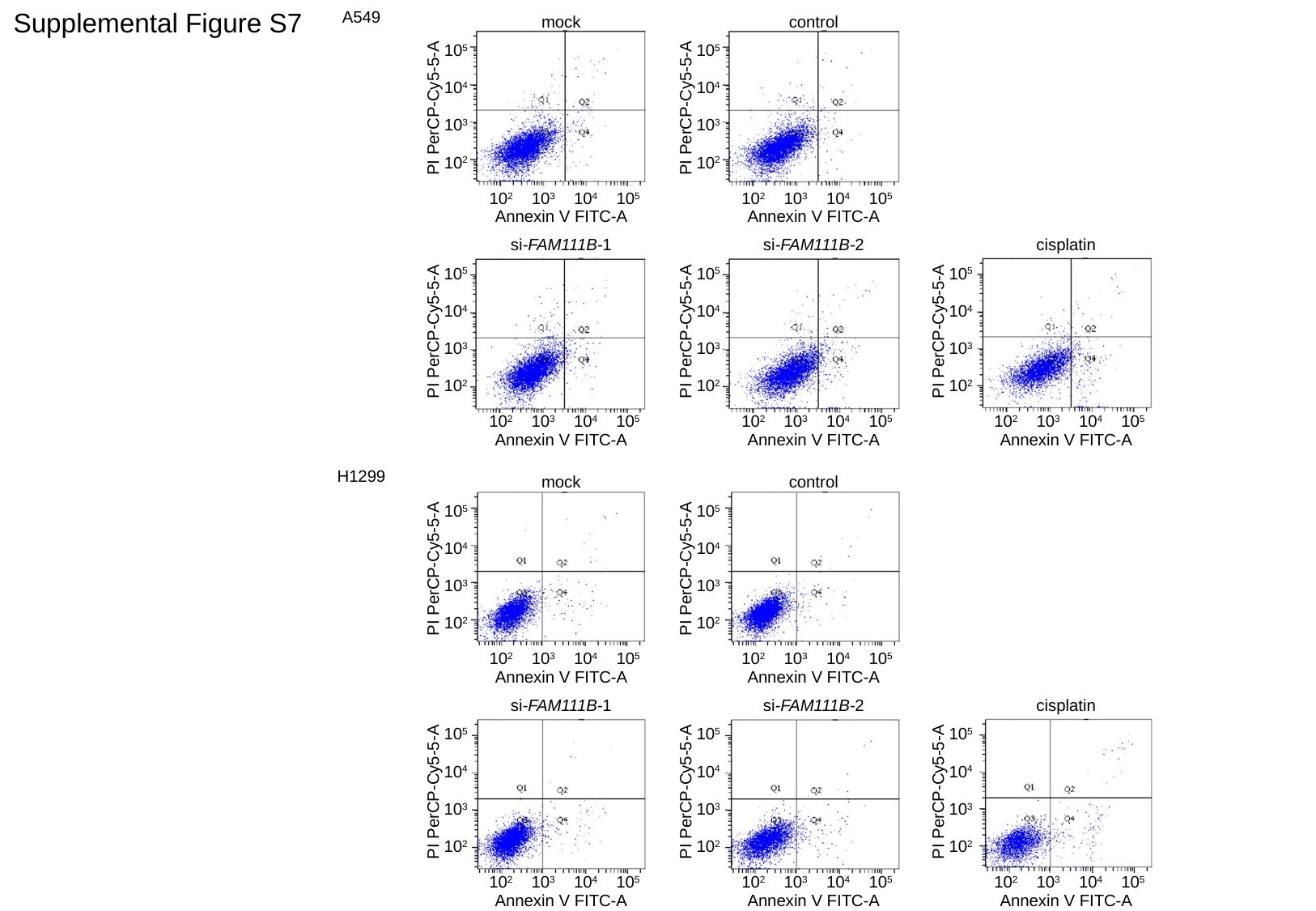

Supplemental Figure S7
A549
mock
105
104
PI PerCP-Cy5-5-A
103
102
102
103
104
105
Annexin V FITC-A
control
105
104
PI PerCP-Cy5-5-A
103
102
102
103
104
105
Annexin V FITC-A
si-FAM111B-1
105
104
PI PerCP-Cy5-5-A
103
102
102
103
104
105
Annexin V FITC-A
si-FAM111B-2
105
104
PI PerCP-Cy5-5-A
103
102
102
103
104
105
Annexin V FITC-A
cisplatin
105
104
PI PerCP-Cy5-5-A
103
102
102
103
104
105
Annexin V FITC-A
H1299
mock
105
104
PI PerCP-Cy5-5-A
103
102
102
103
104
105
Annexin V FITC-A
control
105
104
PI PerCP-Cy5-5-A
103
102
102
103
104
105
Annexin V FITC-A
si-FAM111B-1
105
104
PI PerCP-Cy5-5-A
103
102
102
103
104
105
Annexin V FITC-A
si-FAM111B-2
105
104
PI PerCP-Cy5-5-A
103
102
102
103
104
105
Annexin V FITC-A
cisplatin
105
104
PI PerCP-Cy5-5-A
103
102
102
103
104
105
Annexin V FITC-A
